# Alcohol induces neural tube defects by reducing retinoic acid signaling and promoting neural plate expansion

**DOI:** 10.3389/fcell.2023.1282273

**Published:** 2023-12-05

**Authors:** Tamir Edri, Dor Cohen, Yehuda Shabtai, Abraham Fainsod

**Affiliations:** Department of Developmental Biology and Cancer Research, Institute for Medical Research Israel-Canada, Faculty of Medicine, The Hebrew University of Jerusalem, Jerusalem, Israel

**Keywords:** neural tube closure defects, retinoic acid signaling, fetal alcohol syndrome, *Xenopus* embryo, CRISPR/Cas9, retinol metabolism

## Abstract

**Introduction:** Neural tube defects (NTDs) are among the most debilitating and common developmental defects in humans. The induction of NTDs has been attributed to abnormal folic acid (vitamin B9) metabolism, Wnt and BMP signaling, excess retinoic acid (RA), dietary components, environmental factors, and many others. In the present study we show that reduced RA signaling, including alcohol exposure, induces NTDs.

**Methods:** *Xenopus* embryos were exposed to pharmacological RA biosynthesis inhibitors to study the induction of NTDs. Embryos were treated with DEAB, citral, or ethanol, all of which inhibit the biosynthesis of RA, or injected to overexpress Cyp26a1 to reduce RA. NTD induction was studied using neural plate and notochord markers together with morphological analysis. Expression of the neuroectodermal regulatory network and cell proliferation were analyzed to understand the morphological malformations of the neural plate.

**Results:** Reducing RA signaling levels using retinaldehyde dehydrogenase inhibitors (ethanol, DEAB, and citral) or Cyp26a1-driven degradation efficiently induce NTDs. These NTDs can be rescued by providing precursors of RA. We mapped this RA requirement to early gastrula stages during the induction of neural plate precursors. This reduced RA signaling results in abnormal expression of neural network genes, including the neural plate stem cell maintenance genes, *geminin*, and *foxd4l1.1*. This abnormal expression of neural network genes results in increased proliferation of neural precursors giving rise to an expanded neural plate.

**Conclusion:** We show that RA signaling is required for neural tube closure during embryogenesis. RA signaling plays a very early role in the regulation of proliferation and differentiation of the neural plate soon after the induction of neural progenitors during gastrulation. RA signaling disruption leads to the induction of NTDs through the mis regulation of the early neuroectodermal network, leading to increased proliferation resulting in the expansion of the neural plate. Ethanol exposure induces NTDs through this mechanism involving reduced RA levels.

## Introduction

Malformations of the embryonic neural tube, the precursor of the central nervous system (CNS), are among the most debilitating and common human congenital defects (Finnell et al., 2021; Engelhardt, Martyr, and Niswander, 2022). Worldwide, about 6% of births exhibit a developmental defect (Wallingford et al., 2013), and 19 out of 10,000 live births will present with a neural tube defect (NTD) (Blencowe et al., 2018). NTDs, commonly defined as a failure of neural tube closure (NTC), can develop at any level of the CNS with different extents of severity, outcome, and prevalence (Wallingford et al., 2013; Blencowe et al., 2018; Finnell et al., 2021; Engelhardt et al., 2022). The rarest, craniorachischisis, presents as extensive NTC failure leading to lethality. More prevalent and also lethal is anencephaly, involving severe brain developmental defects. Meningomyelocele (a severe type of *spina bifida*) is restricted mainly to the caudal neural tube and is compatible with survival to adulthood. Multiple mechanisms have been linked to the formation of NTDs including abnormal folic acid (FA; vitamin B9) metabolism, neural tube morphogenesis, cell migration, Wnt/Planar Cell Polarity (PCP) signaling, Bone Morphogenetic Protein (BMP) signaling, retinoic acid (RA) biosynthesis and signaling, in addition to epigenetic abnormalities, exposure to environmental factors, and other etiologies (Harris and Juriloff, 2010; Copp et al., 2013; Wallingford et al., 2013; Zohn, 2020; Finnell et al., 2021; Kakebeen and Niswander, 2021; Engelhardt et al., 2022).

Multiple experimental systems have been developed to study the induction and rescue of NTDs during embryogenesis. Manipulation or mutation of specific genes like *Pax3*, components of the RA and Wnt/PCP signaling pathways, folate metabolic enzymes, and other mutants (mostly in mice) have been used to study the formation of NTDs and their possible prevention (Zhang et al., 2007; Harris and Juriloff, 2010; Wen et al., 2010; Christensen et al., 2013; Momb et al., 2013; Zohn, 2020). In some of these experimental models, the NTDs can be rescued by FA supplementation, whereas others are resistant but can be rescued by other treatments such as inositol or RA (Greene and Copp, 2005; Harris and Juriloff, 2010; D’Souza et al., 2021; Li et al., 2018; Chen et al., 1994; Sudiwala et al., 2016). Multiple reports support maternal dietary fortification with folic acid (FA) to reduce the incidence of NTDs during human development (MRC Vitamin Study Research Group, 1991; Martinez et al., 2021; Kancherla et al., 2022). Although RA can rescue NTDs under specific circumstances, NTD induction by abnormally high RA signaling has been described in multiple studies (Chen et al., 1994; Tonk et al., 2015; Santos-Guzmán et al., 2003; Tom et al., 1991; Barbe et al., 2014; Quemelo et al., 2007; Tran et al., 2011; Kakebeen and Niswander, 2021; Deng et al., 2022; Murphy et al., 2021; Zhang et al., 2007).

The *in utero* exposure of human embryos to alcohol (ethanol, EtOH) induces Fetal Alcohol Spectrum Disorder (FASD) and its common comorbidities, such as craniofacial defects, congenital central nervous system malformations, microcephaly, mental disability, behavioral and social problems, and reduced executive functioning (Popova et al., 2016; Wozniak et al., 2019; May et al., 2022). Individuals suffering from the severe form of FASD, Fetal Alcohol Syndrome (FAS), suffer from numerous additional developmental anomalies, among them also NTDs and brain malformations (Goldstein and Arulanantham, 1978; Peiffer et al., 1979; Wertelecki, 2006; Grewal et al., 2008; De Marco et al., 2011; Popova et al., 2016; Jarmasz et al., 2017; Petrelli et al., 2019). Compared to the worldwide NTD estimate (Blencowe et al., 2018), FAS patients exhibit an increased prevalence of meningomyelocele (1%–2%) and additional spinal cord congenital malformations (12%) (Popova et al., 2016). NTDs have also been reported in alcohol-treated experimental models (Padmanabhan et al., 1994; Coll et al., 2017; Boschen et al., 2021; Randall and Taylor, 1979; Chen et al., 2005; Haghighi Poodeh et al., 2014).

Based on the biochemical similarity between alcohol clearance and RA biosynthesis and the possible overlapping use of enzymes during embryogenesis, the enzymatic competition between both processes has been studied (reviewed in Shabtai and Fainsod, 2018; Fainsod et al., 2022). Multiple developmental malformations characteristic of FAS, such as craniofacial abnormalities, fetal growth restriction, microcephaly, and EtOH-induced abnormal morphogenetic movements, and gene expression changes, were recapitulated by reducing RA signaling levels (reviewed in Fainsod et al., 2022). In agreement, RA has been shown to rescue the teratogenic effects of alcohol exposure in vertebrate embryos (Graham and Ferm, 1985; Wang et al., 2009; Serrano et al., 2010; Ballard et al., 2012; Hwang et al., 2012; Sarmah and Marrs, 2013; Muralidharan et al., 2015; Gupta et al., 2016; Ojeda et al., 2018; Rah et al., 2018; Cadena et al., 2020). Genomic analysis of human NTD patients identified polymorphisms in RA metabolic and signaling network components, some possibly leading to reduced RA signaling that may contribute to this type of developmental malformation (Deak et al., 2005; Tran et al., 2011; Pangilinan et al., 2012; Li et al., 2018; Zou et al., 2020; Wolujewicz et al., 2021a). These observations raise the possibility that RA is required for normal NTC during embryogenesis.

In the present study, we investigated the induction of NTC defects in *Xenopus* embryos by inhibiting the biosynthesis of RA with 4-diethylaminobenzaldehyde (DEAB) or 3,7-dimethyl-2,6-octadienal (citral) (Koppaka et al., 2012; Shabtai et al., 2016). In parallel, we show the induction of NTC defects by alcohol supporting the recent demonstration that acetaldehyde, the oxidation product of EtOH, outcompetes retinaldehyde (RAL) for the available aldehyde dehydrogenase (ALDH) activity in the form of ALDH1A2, resulting in inhibition of RA biosynthesis (Shabtai et al., 2018). Several NTC defect visualization approaches based on notochord labeling to identify it in open neural tubes, or staining the edges of the neural plate to study its closure were developed to facilitate the study of large sample sizes. All three pharmacological inhibitors of RA biosynthesis induced NTC defects with high efficiency. A higher incidence of extensive NTC defects, probably equivalent to craniorachischisis, was observed. Cranial and caudal NTC defects were also observed but with lower prevalence. To further support the reduction of RA by EtOH, supplementation with RA precursors, including retinol (ROL, vitamin A), RAL, or RA itself, rescued the NTC defects induced by alcohol exposure. Early gastrula stages were identified as the most sensitive in the induction of NTC defects by reduced RA signaling. In agreement, more efficient NTC rescue by retinoids was observed when supplementation was provided prior to early gastrulation. Reduced RA signaling resulted in the mis regulation of the neuroectodermal transcriptional network resulting in increased proliferation of the neural plate precursors, and enlarged neural plates. These observations suggest that RA signaling is required for the regulation of neural plate precursor proliferation affecting the subsequent closure of the neural tube and providing a link between abnormal RA signaling and NTC defects.

## Materials and methods

### Embryos and treatments

*Xenopus laevis* frogs were purchased from *Xenopus* 1 or Nasco (Dexter, MI or Fort Atkinson, WI, United States). Experiments were performed after approval and under the supervision of the Institutional Animal Care and Use Committee (IACUC) of the Hebrew University (Ethics approval no. MD-21-16767-3). Embryos were obtained by *in vitro* fertilization, incubated in 0.1% Modified Barth’s Solution and HEPES (MBSH), and staged according to Nieuwkoop and Faber (1967). Embryos were treated with the RA signaling inhibitors; ethanol (0.25%–1.5% vol/vol, Fisher Scientific) 4-Diethylaminobenzaldehyde (DEAB, 60 μM, dissolved in DMSO, Sigma-Aldrich) and 3,7-Dimethyl-2,6-octadienal (citral, 50 μM, diluted in EtOH, Sigma-Aldrich), or with *all-trans* retinol (ROL, 10 μM, Sigma-Aldrich), *all-trans* retinaldehide (RAL, 1 μM, Sigma-Aldrich) and *all-trans* Retinoic Acid (RA, 50 nM, Sigma-Aldrich). Treatments were performed in 0.1% MBSH from the midblastula transition (MBT, NF8.5) until the desired stage for analysis. In the temporal window experiments, embryos were treated at different stages (NF8, NF10.25, NF11, and NF14). All results shown are the summary of at least three full biological replicates where all samples were obtained from a single clutch (batch) of embryos for each replicate.

### RNA injection

Embryos were injected with *in vitro* transcribed capped mRNA of the *cyp26a1* gene (Hollemann et al., 1998). Capped mRNA was prepared from the pCS2 linearized plasmid (cut with NotI, Hollemann et al., 1998) using SP6 RNA polymerase. Cap analog (m7G (5′)ppp (5′)G; New England Biolabs, USA) was added to the reaction mixture using a cap:GTP ratio of 5:1. In the ectoderm-mesoderm germ layer targeting experiment the embryos were injected at the 8-cell stage (NF 4) unilaterally. Using the 16-cell fate map (Moody, 1987a), 100 pg of *cyp26a1* mRNA were injected into the D11-D12 dorsal neural precursor blastomeres and into the dorsal mesoderm precursor blastomeres (D21-D22). The anterior neural tube was targeted by unilaterally injecting 100 pg *cyp26a1* mRNA at 4-cell stage (NF3) to the medial aspect of the dorsal animal cell. For the proliferation analysis, 200 pg *cyp26a1* mRNA was injected into one cell at the 2-cell stage (NF2); these embryos were fixed at NF12 followed by immunohistochemistry and analysis. To analyze the injection site at NTC stages, mRNA solutions were mixed with FITC-dextran. Both NTC and targeted site analyses were performed at NF19-20.

### NTC analysis

The frequency of NTDs was evaluated via morphological examination of the integrity of the neural tube at late neurula (NF19-20). Throughout the experiments, all embryos were kept at the same temperature and conditions. The embryos were divided into four groups by the location and severity of the NTD. Embryos were scored for cranial and caudal NTC problems, extensively open neural tubes along most of their length, and normal-looking embryos. Then, the embryos were counted and fixed for *in situ* hybridization and bisection. The NTD analysis was performed independently by two different researchers.

Whole-mount *in situ* hybridization and double *in situ* hybridization were performed as previously described (Epstein et al., 1997). Embryos were fixed at NF19-20 in MEMFA and processed for whole-mount *in situ* hybridization. Probes were prepared by *in vitro* transcription using digoxigenin or fluorescein labeling nucleotide mixes (Sigma-Aldrich). We performed double whole-mount *in situ* hybridization (dWISH) with sox3 (closing neural tube) (Penzel et al., 1997) together with chrd.1 (notochord) (Sasai et al., 1994). We also employed aquaporin 3 (aqp3) a neural folds marker (Cornish et al., 2009) with pax8, a dynamic pronephros marker (Carroll and Vize, 1999). We also analyzed pax3, a neural plate marker (Bang et al., 1997; Maharana and Schlosser, 2018). Embryos were bisected to confirm open neural tubes.

### Analysis of cell proliferation

Immunofluorescence staining was performed on whole-mount embryos. At the onset of gastrulation, embryos were separated into groups based on the injection side (right or left) based on the FITC-dextran fluorescence, then incubated until late gastrula and fixed for 1–2 h at room temperature in MEMFA [100 mM MOPS (pH 7.4), 2 mM EGTA, 1 mM MgSO_4_, 3.7% (v/v) formaldehyde]. Then, embryos were permeabilized with PBS 1X with 0.1% Triton-X100% and 0.2% BSA solution, incubated in blocking solution (Cas-Block; LIFE technologies; 008120), and stained according to standard procedures. Primary antibody: polyclonal rabbit anti-phospho-Histone H3 (Ser28; 1:500; Upstate 07-145; Sigma-Aldrich). Secondary antibody: Rhodamine 590-conjugated donkey anti-rabbit IgG (1:500, Jackson ImmunoResearch laboratories, 711-295-152). The fluorescence analysis was performed using a Leica M165 FC fluorescence stereo microscope (Leica Microsystems). Image capture was performed with the Leica DFC3000 G monochrome camera controlled with the Leica application suite (LAS) V4.12 software.

### Gene expression analysis by quantitative real-time RT-PCR

Total RNA from embryos was extracted with the Aurum Total RNA Mini Kit (Bio-Rad), and cDNA was synthesized using iScript cDNA Synthesis Kit (Bio-Rad). The real-time PCR reactions were performed using the CFX384 Real-Time System (Bio-Rad) and iTaq universal SYBR Green Supermix (Bio-Rad). Each experiment was repeated at least three independent times and each time the samples were run in triplicate. *slc35b1*.L was used as the housekeeping reference gene (Mughal et al., 2018). The primers used for qPCR analysis are listed in Table 1.

**TABLE 1:** Gene-specific primers used for expression analysis (qPCR).

Gene	Forward primer	Reverse primer
*hoxa1*.L	CCG​CTC​ACT​ATA​TCC​ACC​ATT​C	TGG​CAG​GAG​AAC​GAC​AAA​C
*foxd4l1*.1	TCA​GCA​GCA​AGT​TCC​CTT​AC	CCTGGTTCCCGTGGTATT
*gmnn*	TTG​AAA​GGC​TCA​CTG​GAA​ATG	CTT​CTG​CCA​TGT​CTG​CTT​CA
*sox3*	TTG​GAA​TCT​GTG​TGG​CTG​TT	GGC​TCT​TGA​TGT​CGG​TGT​C
*pax3*.L	AAG​GAA​TGG​TCC​CTC​TTG​TG	GGT​TTG​CTG​TGT​TTC​TGT​CTT​T
*zic1*	CGCCCAACACAGTCTATT	TGTCCGTTCACCACATTG
*cyp26a1*.L	TCGAGGTTCGGCTTCATC	CGGCACAATTCCACAACA
*sox2*	GCA​TGT​CCT​ACT​CCC​AAC​AAG	GGG​AAG​AAG​AGG​TGA​CTA​CAG​G
*msx1*.L	CAA​ACT​GGC​CTG​AAG​ATG​TCC​C	CAG​TTG​GGC​TGG​ACC​TAT​CTC​TC
*bmp4*	CTT​CTG​TGC​CTG​GTA​GAT​TC	GCGCCCAGTAAGGATGT
*krt12.4*	ATG​GCA​GAA​GCA​GCA​AGT​T	CTT​TAG​ACC​CAG​CAC​CAA​TG
*slc35b1*.L	CGC​ATT​TCC​AAA​CAG​GCT​CC	CAA​GAA​GTC​CCA​GAG​CTC​GC

### Statistical analysis

All experiments were repeated at least three times analyzing at least three independent clutches of embryos. For statistical analysis we employ the Prism software (GraphPad) to perform ANOVA, *t*-test or Fisher’s exact test depending on the type of data distribution.

## Results

### Reduced RA signaling induces NTDs

**FIGURE 1:**
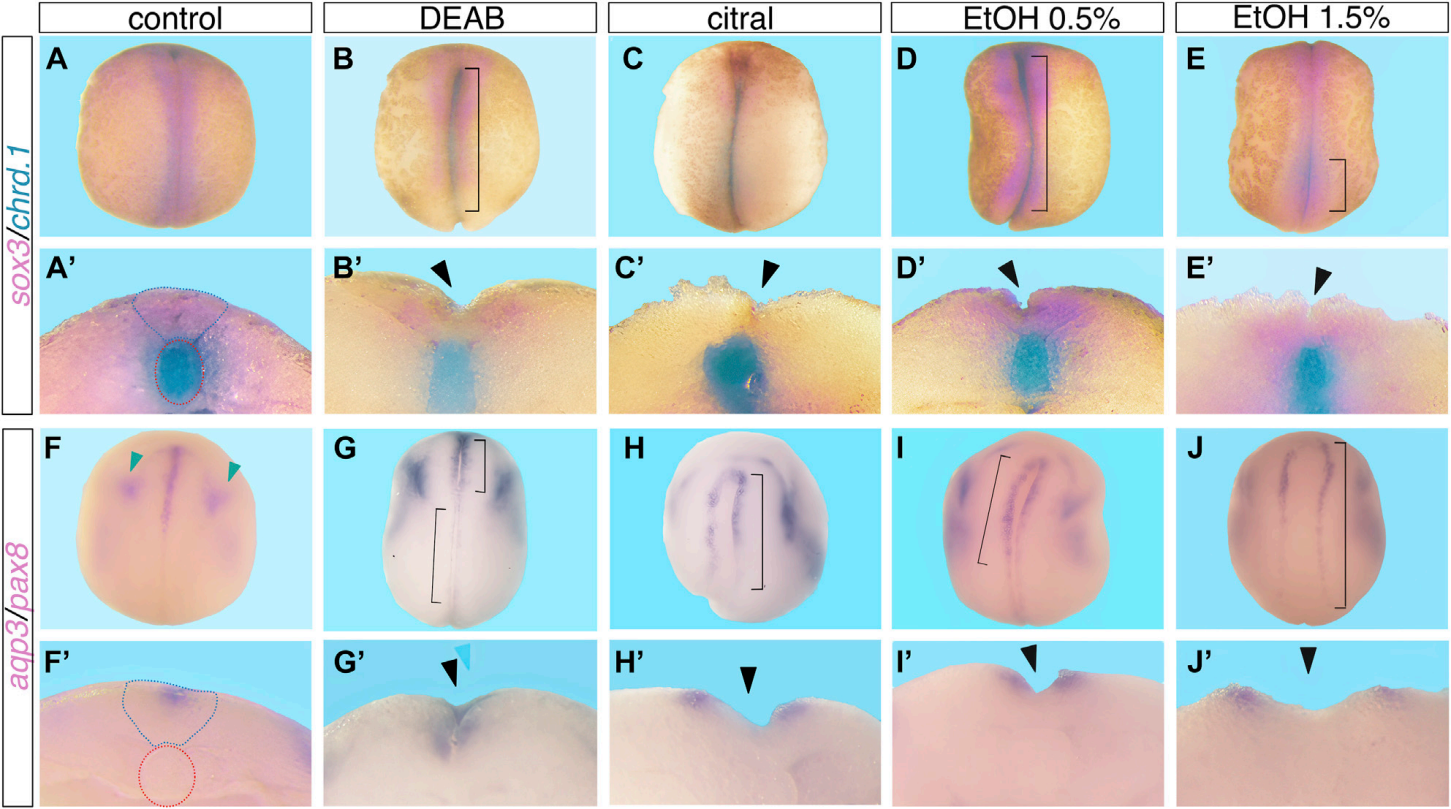
Reduced RA signaling induces neural tube closure defects. Embryos were treated with DEAB (60 µM), citral (50 µM), or EtOH (0.5% or 1.5% vol/vol) from mid-blastula stages (NF8), and were allowed to develop to neural tube closure stages (NF 19-20). Experimental and control embryos were processed for whole-mount *in situ* hybridization with markers of the neural plate and notochord, **(A–E’)** *sox3* and *chrd.1* and **(F–J’)** *aqp3* and *pax8*. **(A–E)**, **(F–J)** Dorsal view of embryos, anterior to the top. **(A’-E’)**, **(F’J’)** Cross section of the same embryo shown in the panel above, dorsal to the top. Brackets mark NTC defects, black arrowheads mark open neural tubes, blue dotted outline marks the closed neural tube, red dotted outline around the notochord, and green arrowheads mark the *pax8* expression.

**FIGURE 2:**
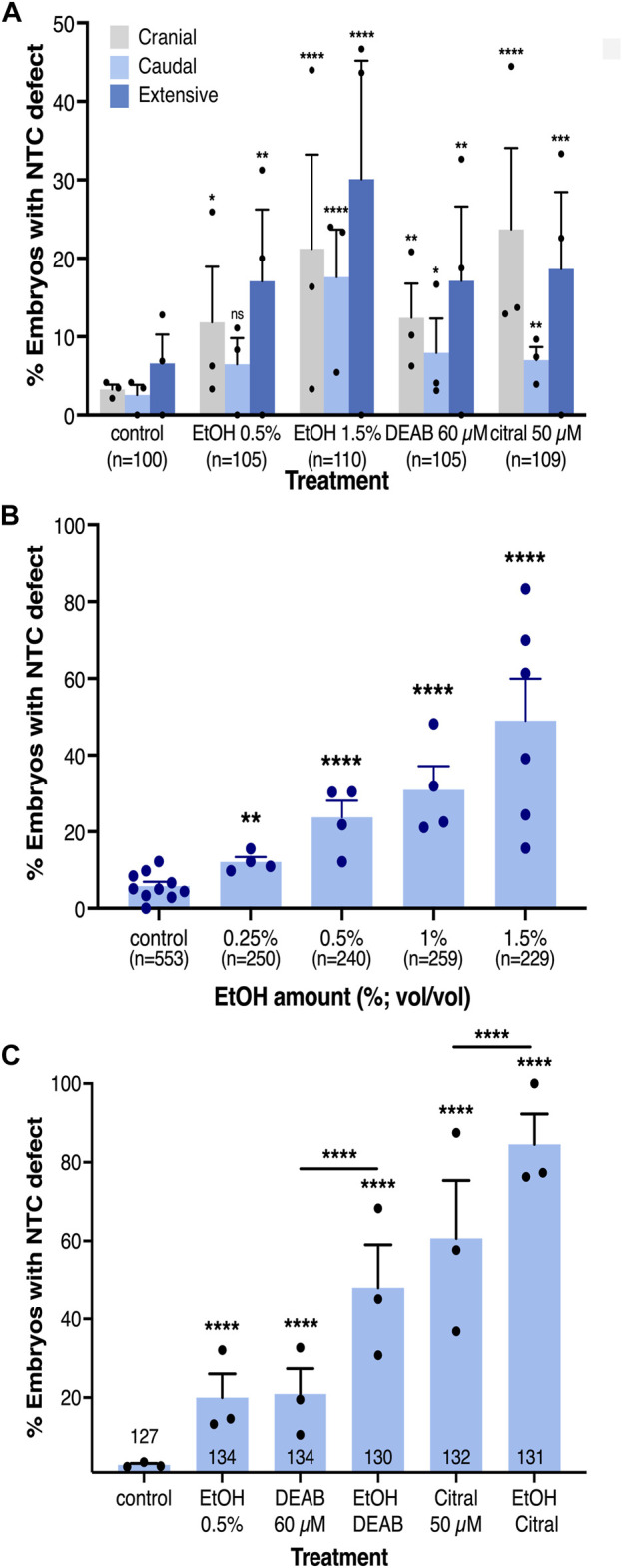
The incidence of NTC defects at different axial levels by reduced RA signaling is concentration-dependent. **(A)** Analysis of the rostral-caudal distribution of NTDs with the different pharmacological inhibitors of RA biosynthesis. **(B)** EtOH concentration dependence in the induction of NTC defects. **(C)** Additive effect of combinations of RA biosynthesis inhibitors. Embryos were treated with DEAB (60 µM), citral (50 µM), or EtOH (0.5%) alone or in combination and subsequently analyzed for NTC defect induction. The percent of embryos with a NTC defect is shown. Sample sizes are shown as well as the distribution of the biological replicates analyzed. *, *p* < 0.05; **, *p* < 0.01; ***, *p* < 0.001 ****, *p* < 0.0001; ns, not significant. Lines denote samples compared; no line, compared to the control sample.

To establish an experimental model to study the involvement of RA signaling in the closure of the neural tube, we exposed *Xenopus* embryos to RA biosynthesis inhibitors. We treated embryos with 3,7-dimethyl-2,6-octadienal (citral) or 4-diethylaminobenzaldehyde (DEAB) to inhibit the biosynthesis of RA from retinaldehyde (RAL) performed by the main retinaldehyde dehydrogenases (ALDH1A1, ALDH1A2, and ALDH1A3) (Boyer and Petersen, 1991; Mahmoud et al., 1993; Koppaka et al., 2012; Shabtai et al., 2016). Embryos were treated with DEAB (60 µM) or citral (50 µM) from mid blastula stage (NF8) until neural tube closure stages (NF19-20) (Nieuwkoop and Faber, 1967: NF) and subsequently subjected to NTC analysis. Several approaches and molecular probes were used to analyze the differentiation and closure of the neural tube. We used *sox3* expression to mark the closing neural tube (Penzel et al., 1997) in conjunction with *chrd.1* (formerly *chordin*) to label the notochord (Sasai et al., 1994) (Figure 1). Using double whole-mount *in situ* hybridization (dWISH) we identified NTDs by scoring whether the notochord (blue) signal was visible from a dorsal view in open neural tubes compared to control, closed neural tubes (Figures 1A–C’). We also employed *aquaporin 3* (*aqp3*) (Cornish et al., 2009) as a marker of the neural folds jointly with the pronephros marker, *pax8* to monitor the developmental stage of the treated embryos (Carroll and Vize, 1999) (Figure 1F–H’). We also employed *pax3*, a very dynamic marker of neural plate differentiation and morphogenesis (Bang et al., 1997; Maharana and Schlosser, 2018) to aid in scoring NTC defects (Supplementary Figure S1A–C’). NTC defects were confirmed by bisecting embryos and scoring for open neural tubes. In the reduced RA embryos we observed the major NTC types commonly described in studies focused on humans or experimental animal models (Northrup and Volcik, 2000; Wallingford et al., 2013; Engelhardt et al., 2022; Isaković et al., 2022). We observed NTC problems in the cranial region (Figures 1G, 2A; Supplementary Figure S1C) probably equivalent to anencephaly. NTC defects restricted to the caudal neural tube, equivalent to meningomyelocele, were also observed (Figures 1G, 2A). The more severely affected embryos exhibited extensively open neural tubes along most of their length, reminiscent of craniorachischisis (Figures 1B, C, H, Figure 2A; Supplementary Figure S1B). In some instances, bilateral or unilateral loss of neural plate marker expression was observed (Figure 1H). Treatment with both RA biosynthesis inhibitors resulted in efficient NTC defect induction reaching about 39% affected embryos exposed to DEAB and 50% of embryos treated with citral (Figure 2A). These results show that pharmacological inhibition of RA biosynthesis results in NTC defects at all levels of the neural tube.

Our previous studies showed that EtOH exposure induces developmental malformations recapitulating the FAS phenotype by inhibiting RA biosynthesis and reducing RA signaling (Shabtai et al., 2018; Fainsod et al., 2022). Therefore, we also employed EtOH as an additional inhibitor of RA production to further corroborate the involvement of RA signaling in the NTC process and the induction of closure defects. *Xenopus* embryos were treated with EtOH (0.5% or 1.5% vol/vol) from mid-blastula (NF8) to neural tube closure stages (NF19-20). Analysis of abnormal neural tube closure was performed as above (Figures 1D, E, I, J; Supplementary Figures S1D, E). We observed NTC defects in the cranial region (Figure 2A), and in the caudal neural tube (Figures 1E, 2A). As observed with DEAB and citral treatment, the more severely affected embryos and the more common phenotype presented as extensively open neural tubes along most of their length (Figures 1D, J’, Figure 2A; Supplementary Figure S1E). We also occasionally observed unilateral loss of neural plate marker expression (Figure 1I; Supplementary Figure S1E). These results show that EtOH exposure, like the other inhibitors of RA biosynthesis, efficiently induces NTDs.

Our analysis of the rostral-caudal distribution of the NTC defects as a result of embryonic alcohol exposure revealed an increase in the incidence of each type NTC defect at the higher EtOH concentration (1.5%; Figure 2A). To better understand the difference between responses to low and high EtOH concentrations, we treated embryos with multiple alcohol concentrations from 0.25% to 1.5% for NTC defect analysis (Figure 2B). We observed a clear concentration-dependent response in the induction of NTC defects by EtOH. At the lowest (0.25%) EtOH concentration 12% of the embryos exhibited NTDs while at 1.5% EtOH, 61% of the embryos had NTC defects (Figure 2B). These results show that EtOH exposure efficiently induces NTDs in *Xenopus* embryos.

### EtOH induces NTDs by reducing RA signaling levels

The induction of NTC defects by EtOH with efficiencies and outcomes resembling the effect of well-characterized RA biosynthesis inhibitors (DEAB and citral) supports the effect of EtOH as an inhibitor of RA production (Shabtai et al., 2018). To further support the EtOH-promoted reduction in RA levels in the induction of NTC defects, we studied whether alcohol sensitizes the embryo to RA biosynthesis inhibition by treating embryos with alcohol together with either DEAB or citral. The individual treatments with all three RA biosynthesis inhibitors resulted in NTC defect incidences similar to the initial *in situ* hybridization-based analysis (Figures 2A, C). Combined treatment of DEAB or citral with a low concentration of EtOH (0.5%) resulted in a significant additive effect, increasing the incidence of NTC defects irrespective of the inhibitor employed (Figure 2C). The NTD incidence for DEAB alone (20.9%) or EtOH alone (19.9%) increased to 48.1% affected embryos when they were supplied in combination. Similarly, NTDs induced by citral increased from 60.6% when provided alone, to 84.5% when combined with EtOH. These results show that reducing the levels of RA signaling hypersensitized the embryo to alcohol exposure.

**FIGURE 3:**
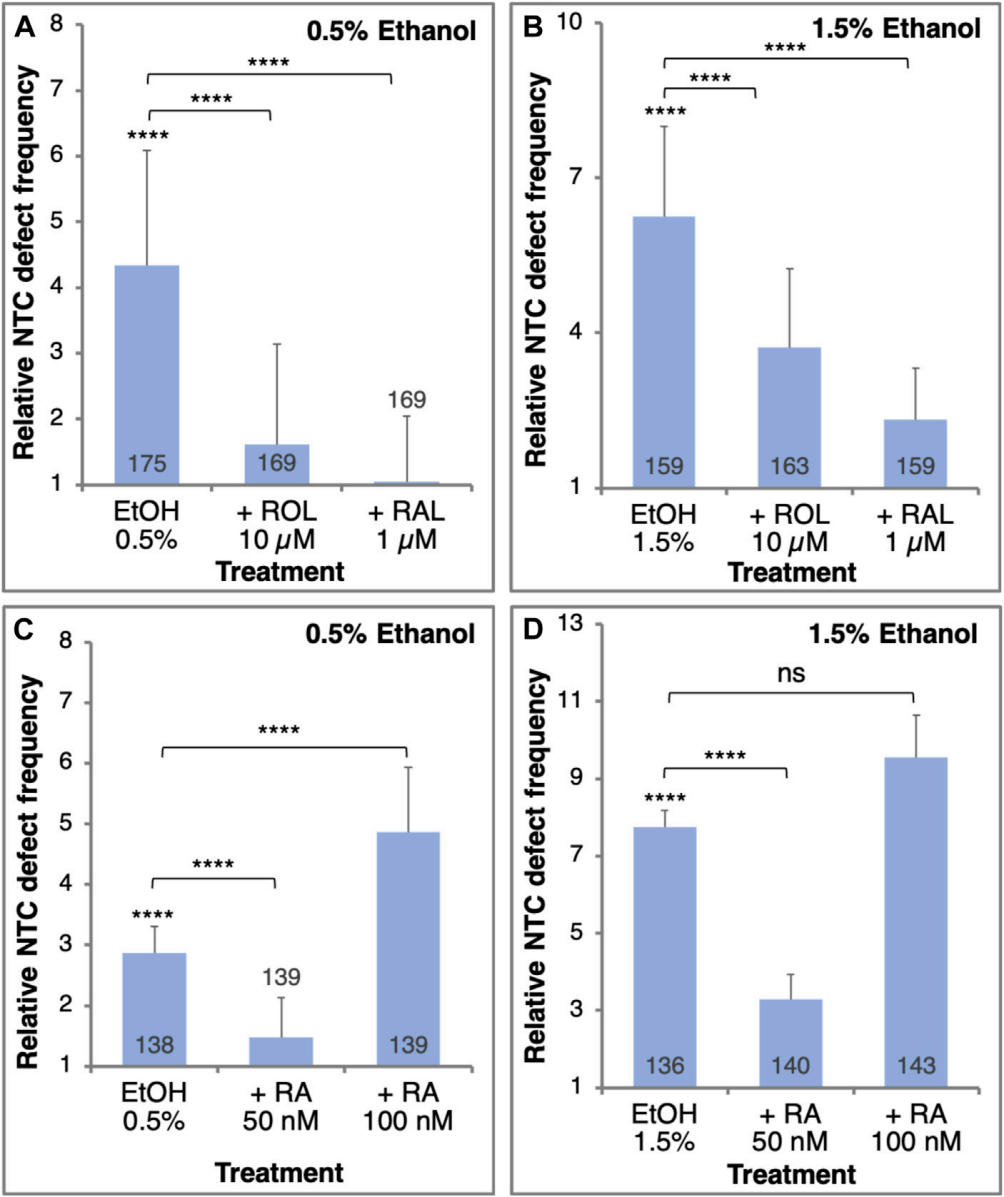
The EtOH-induced NTC defects can be rescued by the addition of RA or its precursors. Embryos were treated with 0.5% **(A,C)** or 1.5% **(B,D)** EtOH to induce NTC defects. Together with the EtOH treatment, the embryos were supplemented with retinol (ROL, 10 µM), retinaldehyde (RAL, 1 µM) (A, B), or RA (50 nM or 100 nM) (C, D). The frequency of NTC defects was calculated relative to control samples. Sample sizes are shown inside each bar. ****, *p* < 0.0001; ns, not significant. Brackets denote samples compared, no bracket, compared to the control sample.

To demonstrate whether EtOH promotes NTC defects through a reduction in RA signaling, we performed a series of rescue experiments. NTC defects were induced by two EtOH concentrations (0.5% and 1.5%) and were rescued by providing RA or its precursors, retinol (vitamin A, ROL) or retinaldehyde (RAL) (Figure 3). Irrespective of the amount of EtOH exposure, ROL or RAL significantly reduced the incidence of NTC defects (Figures 3A, B). The extent of NTC rescue is inversely related to the amount of EtOH used to treat the embryos. A similar rescue effect was observed when embryos were treated with 50 nM RA together with EtOH at either concentration (Figures 3C, D). Increasing the amount of the RA to 100 nM resulted in an increase in NTC defects instead of a rescue effect (Figures 3C, D), recapitulating reports showing that excess RA can induce NTDs through an unknown mechanism (Alles and Sulik, 1990; Abu-Abed et al., 2001; Kakebeen and Niswander, 2021; Maassel et al., 2021). Then, in agreement with the suggested etiology of FASD involving a teratogenic reduction in RA (Shabtai et al., 2018), EtOH also induces NTC defects by the same mechanism and they are significantly rescued by adding low levels of RA or its precursors.

In previous studies involving reduced RA signaling and alcohol exposure, we described a developmental delay during gastrula stages and the recovery from this delay by early neurula stages (Yelin et al., 2005; Yelin et al., 2007; Gur et al., 2022a). To rule out the possibility that a developmental delay is the reason for the observation of a high proportion of embryos with NTC defects following alcohol exposure, we performed a detailed analysis of the progression through embryogenesis of EtOH-treated embryos. Groups of embryos were treated with low (0.5%) or high (1.5%) EtOH concentrations starting at mid-blastula (NF8), early gastrula (NF10.25), or mid-gastrula (NF11). The NF stage of each embryo was assigned when the control sibling embryos reached late gastrula/early neurula (NF13). The same embryos, as well as an additional group treated from early neurula (NF14), were further incubated until the control embryos reached NTC stages (NF19) at which time staging was again determined for all embryos (Supplementary Figure S2). Analysis of the alcohol-treated embryos at late gastrula/early neurula stages revealed a delay in the progression through gastrula that became more pronounced the earlier the treatment was initiated and the higher the concentration of EtOH employed (Supplementary Figure S2A). The same embryos analyzed during NTC stages exhibited exactly the same stage distribution as their control siblings irrespective of the level or timing of the EtOH treatment (Supplementary Figure S2B). These results were further supported by analysis of the *pax8* expression pattern which has a very dynamic and informative expression pattern around NF19 (Heller and Brändli, 1999), indicating that experimental and control embryos are at approximately the same developmental stage during NTC stages (Figures 1F–J’). These data demonstrate, as previously described, that the reduction of RA signaling by EtOH exposure induces a transient developmental delay that is overcome during early neurula stages (Yelin et al., 2007; Gur et al., 2022b), and the NTDs observed in the EtOH-treated embryos are not the result of a developmental delay.

To confirm the correct staging of embryos during NTC stages (NF19 or NF20), we focused on morphological landmarks independent of the nueral folds. In their normal table of *Xenopus laevis* Nieuwkoop and Faber (1967) describe changes in the shape and size of the body. These stage-specific morphological changes were reiterated in the new description of *Xenopus* development (Zahn et al., 2022). To confirm these staging criteria in reduced RA-treated embryos, we focused on the changing shape of the embryo, particularly, elongation. The length and width of previously staged, EtOH-treated and control embryos were measured. To normalize for genetically driven size variability (Leibovich et al., 2020), we calculated the length-to-width ratio of each embryo (Supplementary Figure S2C). Irrespective of whether treated or control, the length-to-width ratio was characteristic for NF19 or NF20 and significantly different from each other. These observations further confirm the annulment of the developmental delay by NTC stages and the morphological staging criteria employed.

### Reduced RA during late blastula/early gastrula induces NTDs

**FIGURE 4:**
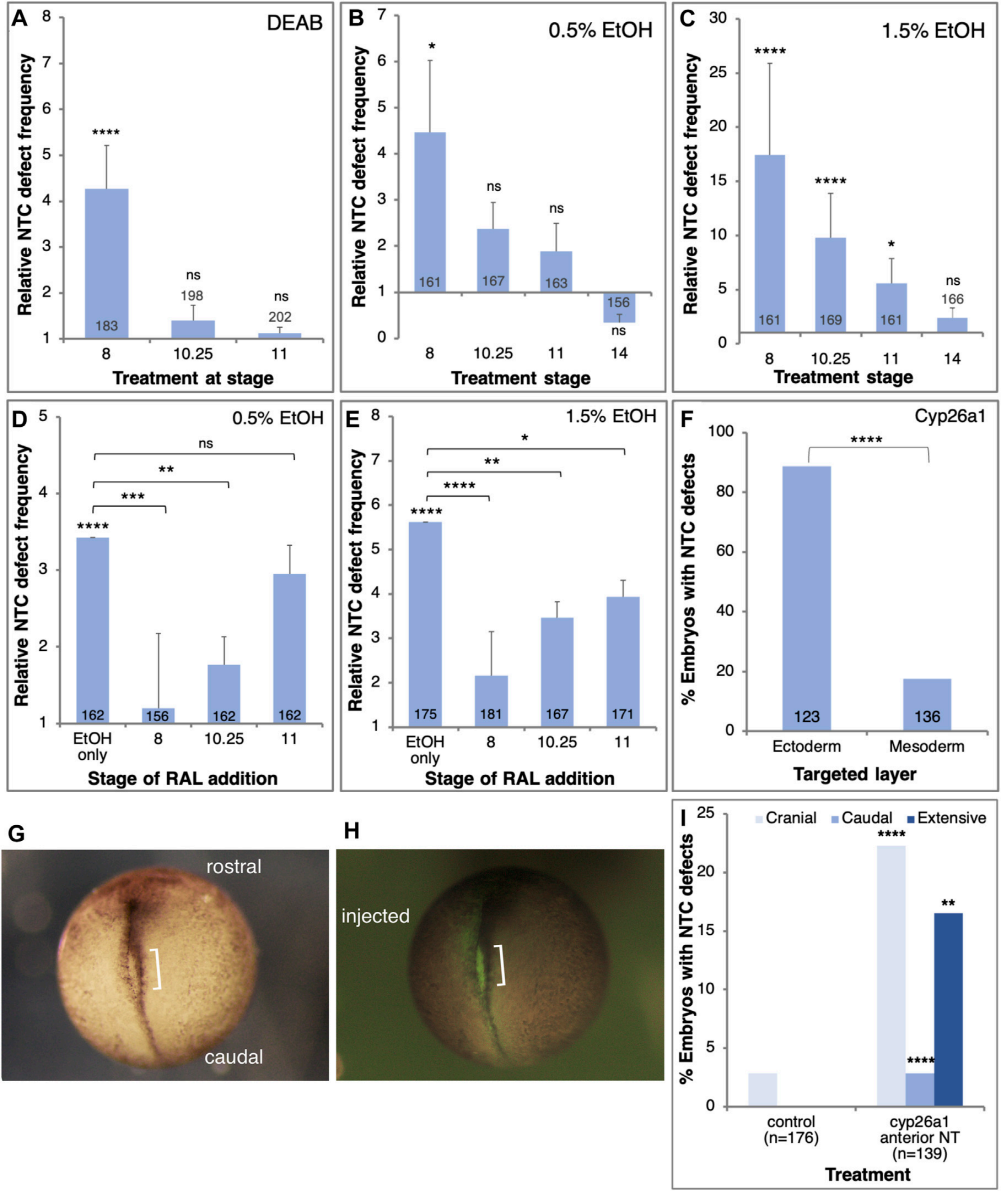
RA signaling is required in the prospective ectoderm at the onset of gastrulation to prevent NTC defects. **(A–C)** Embryos were exposed to DEAB **(A)**, 0.5% EtOH **(B)**, or 1.5% EtOH **(C)**, starting at different developmental stages (NF8, 10.25, 11, 14). The frequency of NTC defects was calculated relative to control samples. **(D,E)** Embryos were treated with 0.5% **(D)** and 1.5% EtOH **(E)** to induce NTC defects. RAL (1 µM) was then added at different stages to rescue the reduction in RA levels. **(F)** Embryos were injected with mRNA encoding the Cyp26a1 enzyme. Injections targeted unilaterally either the prospective neuroectoderm (B-tier) or dorsal mesoderm (C-tier). The injection site was corroborated by the co-injection of FITC-dextran. **(G–I)** Embryos were injected with *cyp26a1* mRNA targeting the prospective anterior neural plate. FITC-dextran was co-injected to label the injection site. At NTC stages (NF19) the embryos were monitored for NTC defect induction, the localization of the NTC defects, and the overlap with the lineage tracer. The orientation of the embryos, the injected side, and the NTD are shown. Sample sizes are shown within each bar. *, *p* < 0.05; **, *p* < 0.01; ***, *p* < 0.001; ****, *p* < 0.0001; ns, not significant. Brackets denote samples compared, no bracket, sample compared to the control sample.

Previously we showed that the embryo is most sensitive to alcohol exposure close to the onset of gastrulation (Yelin et al., 2005; Gur et al., 2022a; Gur et al., 2022b). In order to determine the sensitivity window for NTC defect induction by reduced RA signaling, we treated embryos with either EtOH or DEAB starting the treatments at different developmental stages. NTC defect analysis was performed at NF19-20. Reducing RA production by DEAB treatment resulted in significant NTC defect induction when initiated during mid-blastula stages (NF 8; Figure 4A). Reducing RA biosynthesis from early gastrula (NF10.25) or later resulted in no significant induction of NTC defects (Figure 4A). Similarly, exposure to low EtOH concentrations (0.5%) revealed a significant level of NTC defects when the treatment was started during blastula stages (NF8; Figure 4B). Starting the 0.5% EtOH treatment during gastrula stages resulted in weaker, and not significant NTC defect induction (Figure 4B). Treatment of embryos with higher (1.5%) EtOH concentrations resulted in a similar pattern of NTC defect induction (Figure 4C). Also 1.5% EtOH efficiently induced NTC defects when added during blastula stages (Figure 4C), while alcohol addition during early gastrula (NF10.25) resulted in weaker NTC defect induction efficiency that continues to decrease the later the treatment is initiated (Figure 4B). These results show that reducing RA signaling induces NTC defects by interfering with a developmental process taking place during late blastula/early gastrula stages.

To better map the NTD induction sensitivity window we performed rescue experiments at different developmental stages. *Xenopus* embryos were treated with low or high EtOH concentrations at mid-blastula (NF8), and RAL was added at different developmental stages to restore RA signaling levels (Figures 4D, E). In agreement with the inhibition of RA biosynthesis experiments, irrespective of the amount of EtOH employed, the most efficient NTC defect rescue was observed when RAL was added during mid-blastula stages (Figures 4D, E). RAL addition during early gastrula (NF10.25) resulted in a less efficient although significant rescue of the NTC defects. The NTC defect rescue efficiency decreased as the embryo progressed through gastrulation. These results show that the RA levels must be restored by late blastula/early gastrula to efficiently rescue the induction of NTC defects by EtOH.

To identify the cells most sensitive to the RA reduction in the induction of NTC defects we reduced the levels of RA in a localized manner by injecting mRNA encoding the RA-hydroxylase, Cyp26a1 (Ross and Zolfaghari, 2011). This enzyme targets RA for degradation thus reducing RA signaling (Ribes et al., 2007; Thatcher and Isoherranen, 2009). Taking advantage of the available lineage maps (Dale and Slack, 1987; Moody, 1987b; Klein, 1987), *cyp26a1 m*RNA was injected on the prospective dorsal side of the B-tier to target the future neuroectoderm (B1) or the C-tier to target the prospective axial mesodermal cells (C1). Injections were performed at the 8- or 16-cell stage to achieve a more extensive mRNA distribution. The *cyp26a1* RNA was co-injected with FITC-dextran to confirm the injection site. The injected embryos were incubated to NTC stages and the extent of closure defect induction was determined. Targeting the future neuroectodermal cells resulted in more than a four-fold higher incidence of NTC defect induction than targeting the prospective axial mesoderm (Figure 4F). These results show that experimental reduction of RA signaling targets an RA-dependent process in the prospective neuroectoderm early during gastrulation that results in the induction of NTC defects.

To spatially link the reduction of RA levels and the induction of NTC defects, we injected embryos with *cyp26a1* mRNA targeting the anterior neural plate. Embryos were analyzed for the anterior-posterior position of the NTC defects and the overlap with the FITC-dextran lineage tracer (Figures 4G, H). Compared to the systemic inhibition of RA biosynthesis (EtOH, DEAB, and citral; Figure 2A), the localized overexpression of Cyp26a1 in the cranial region enhanced the incidence of NTC defects in this domain (Figure 4I). These results demonstrate a significant correlation between the site of RA reduction and the site of NTC defect induction, indicating that defects are induced in neural plate areas that experience reduced RA levels.

### Reduced RA signaling hampers neural differentiation

**FIGURE 5:**
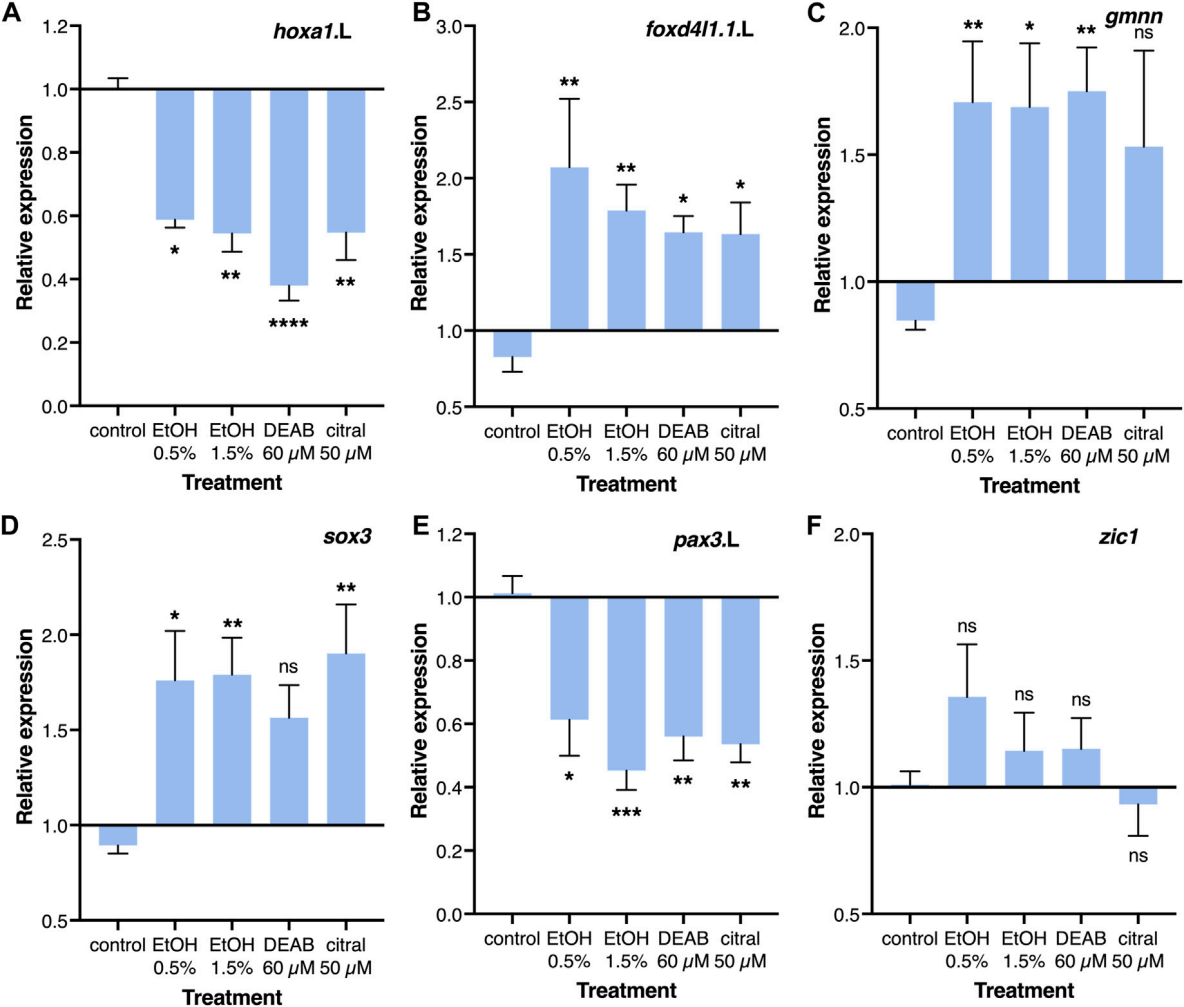
Regulation of the neuroectodermal regulatory network by RA. Embryos were treated with EtOH (0.5% and 1.5%), DEAB (60 µM), or citral (50 µM) from the midblastula stage (NF8) and incubated until early/mid gastrula (NF10.5) for qPCR analysis. **(A–F)** Relative expression for *hoxa1*.L, *foxd4l1.1*.L, *gmnn*, *sox3*, *pax3*.L, and *zic1*, respectively. Expression levels were normalized to the control expression level at NF 10.5. *, *p* < 0.05; **, *p* < 0.01; ***, *p* < 0.001; ****, *p* < 0.0001; ns, not significant. Samples compared to the control sample.

The effects of RA reduction on the incidence of NTC defects, and in particular the late blastula/early gastrula sensitivity of this effect, suggest interference with a regulatory interaction taking place at these early developmental stages. The initial steps in the induction and differentiation of the neuroectoderm take place during early gastrulation and the manipulation of this process can affect proliferation, cell death, differentiation, morphogenesis, and additional events required for the normal formation of a neural tube (Moody et al., 2013; Klein and Moody, 2015; Sankar et al., 2016; Sherman et al., 2017). Therefore, a reduction in RA signaling that interferes with a very early process in neural differentiation could trigger the induction of NTC defects. For these reasons, we studied the transcriptional effect of RA reduction on the early neuroectodermal regulatory network (Moody et al., 2013). Embryos were treated from late blastula with EtOH, DEAB, or citral, and RNA samples were collected during early/mid gastrula (NF10.5) for quantitative real-time RT-PCR (qPCR) analysis. The efficiency of the treatments was monitored by analyzing the expression of the known RA-regulated genes, *hoxa1.*L and *cyp26a1.*L which exhibited significant reductions of about 40% and 30%, respectively (Figure 5A; Supplementary Figure S3A).

Two early regulators of the early neuroectodermal network, *foxd4l1.1* (formerly *foxd5*) and *geminin* (*gmnn*) have been shown to maintain the neuroectodermal precursors in a committed state while allowing their proliferation and preventing differentiation (Moody et al., 2013; Sankar et al., 2016). The reduction of RA levels resulted in a significant increase in *foxd4l1.1* (1.63-2.07 fold) and *gmnn* expression *(*1.53-1.75 fold) depending on the treatment (Figures 5B, C). Analysis of neural plate stem cell genes revealed an increase in *sox3* expression (56%–90%; Figure 5D), whereas *zic1, sox2,* and *sox11* exhibited no significant changes in their expression levels (Figure 5F; Supplemental Figures S3B, S3C). The neural plate border genes exhibited significant downregulation following the RA reduction treatments, *pax3* (39%–65%) and *msx1* (23%–42%) (Figure 5E; Supplementary Figure S3D). The epidermal markers *bmp4* and *krt12.4* both exhibited weak responses showing no significant effect of reduced RA on these ectodermal derivatives (Supplemental Figures S3E, S3F). These gene expression changes suggest that RA signaling plays an important regulatory role in the transition from the maintenance and proliferation of neuroectodermal precursors to the early stages of neural differentiation, probably leading to overproliferation and not the initial neural and epidermal differentiation of the ectoderm.

**FIGURE 6:**
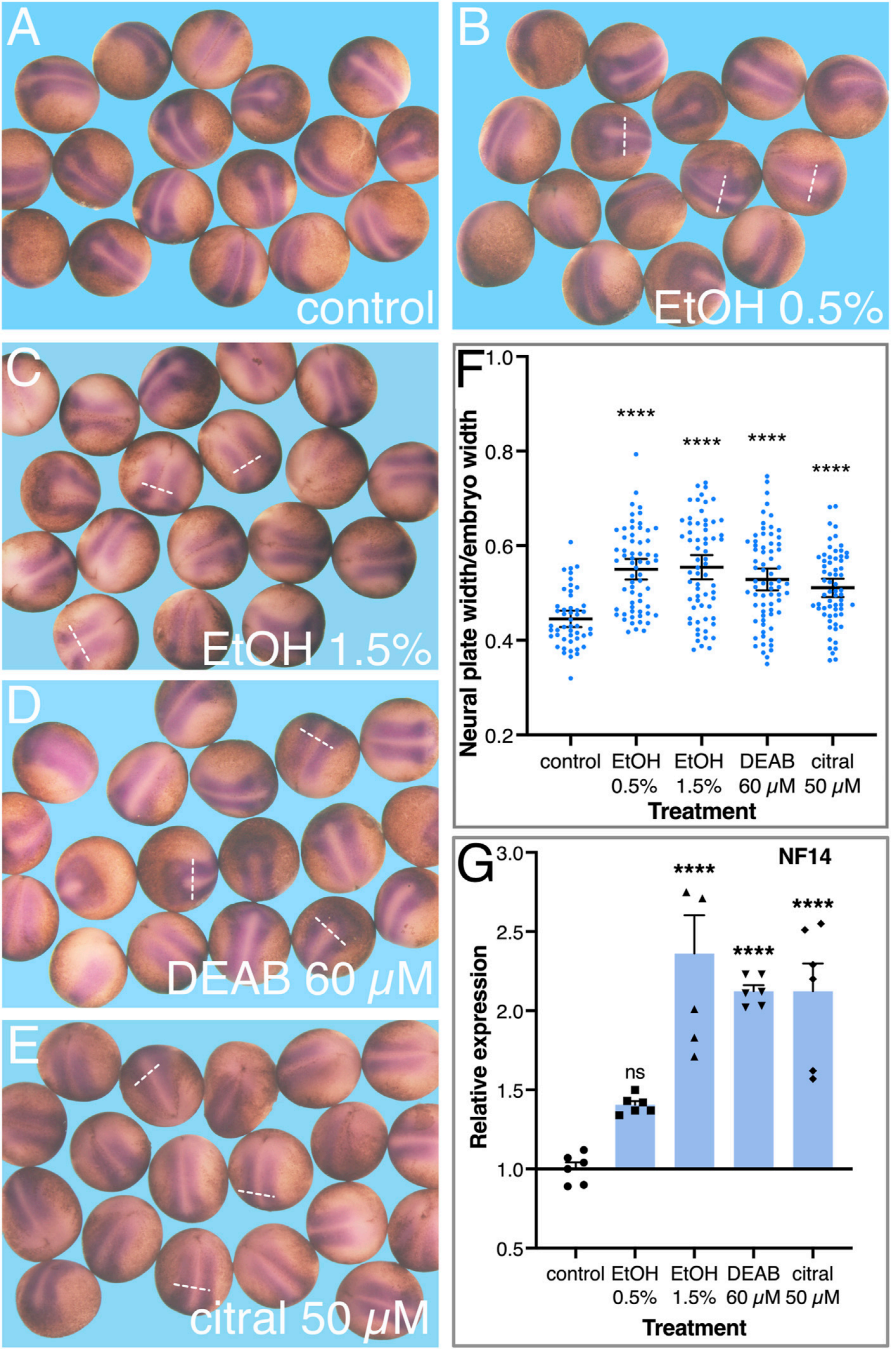
Reduced RA signaling results in the expansion of the neural plate. Embryos were treated with EtOH (0.5% and 1.5%), DEAB (60 µM), and citral (50 µM) and collected for analysis during early neurula stages (NF 14). **(A–E)** The neural plate was visualized by *in situ* hybridization with the early marker, *sox3*. **(F)** The width of the neural plate was measured in all experimental samples and normalized to the overall width of the embryo to account for individual size changes. **(G)** The changes in *sox3* expression levels were determined by qPCR with gene-specific primers. *, *p* < 0.05; ***, *p* < 0.001; ****, *p* < 0.0001; ns, not significant. Samples compared to the control sample.

To better characterize the early changes in the neural plate as a result of RA signaling knockdown, embryos were treated with EtOH, DEAB, or citral, incubated to early neurula stages (NF14), and processed for *in situ* hybridization with a *sox3*-specific probe to study the development of the neural plate. Comparison of the *sox3* expression domain in treated and control embryos suggested an expansion of the neural plate irrespective of the RA-reducing treatment (Figures 6B–E). To quantitate the extent of neural plate expansion along the medial-lateral axis, the width of the *sox3* expression domain was measured just caudal to the hindbrain, together with the overall width and length of each embryo. To account for changes in embryo sizes, even within a single clutch (Leibovich et al., 2020), the width of the neural plate (i.e., the *sox3* expression domain) was normalized to the total width of the embryo (Figure 6F). These measurements revealed a significant expansion of the width of the neural plate as a result of the reduction in RA levels by each of the four treatments (Figure 6F). We also normalized the neural plate width to the embryo length with very similar results (not shown), a medial-lateral expansion of the neural plate. To further corroborate this neural plate expansion, we determined the relative expression level of *sox3* at NF14 as a result of the reduction of RA signaling. By NF14, expression of *sox3* in the DEAB, citral, or EtOH-treated embryos increased by 42%–238% (Figure 6G). This observation further supports an expansion of the neural plate and is in agreement with the continued or enhanced proliferation of neuroectodermal precursors. A similar expression analysis performed during early/mid gastrula (NF10.5) revealed an early upregulation of *sox3* (Figure 5D) suggesting the increase in expression and the subsequent expansion of the neural plate are the outcomes of events taking place during early gastrula.

**FIGURE 7:**
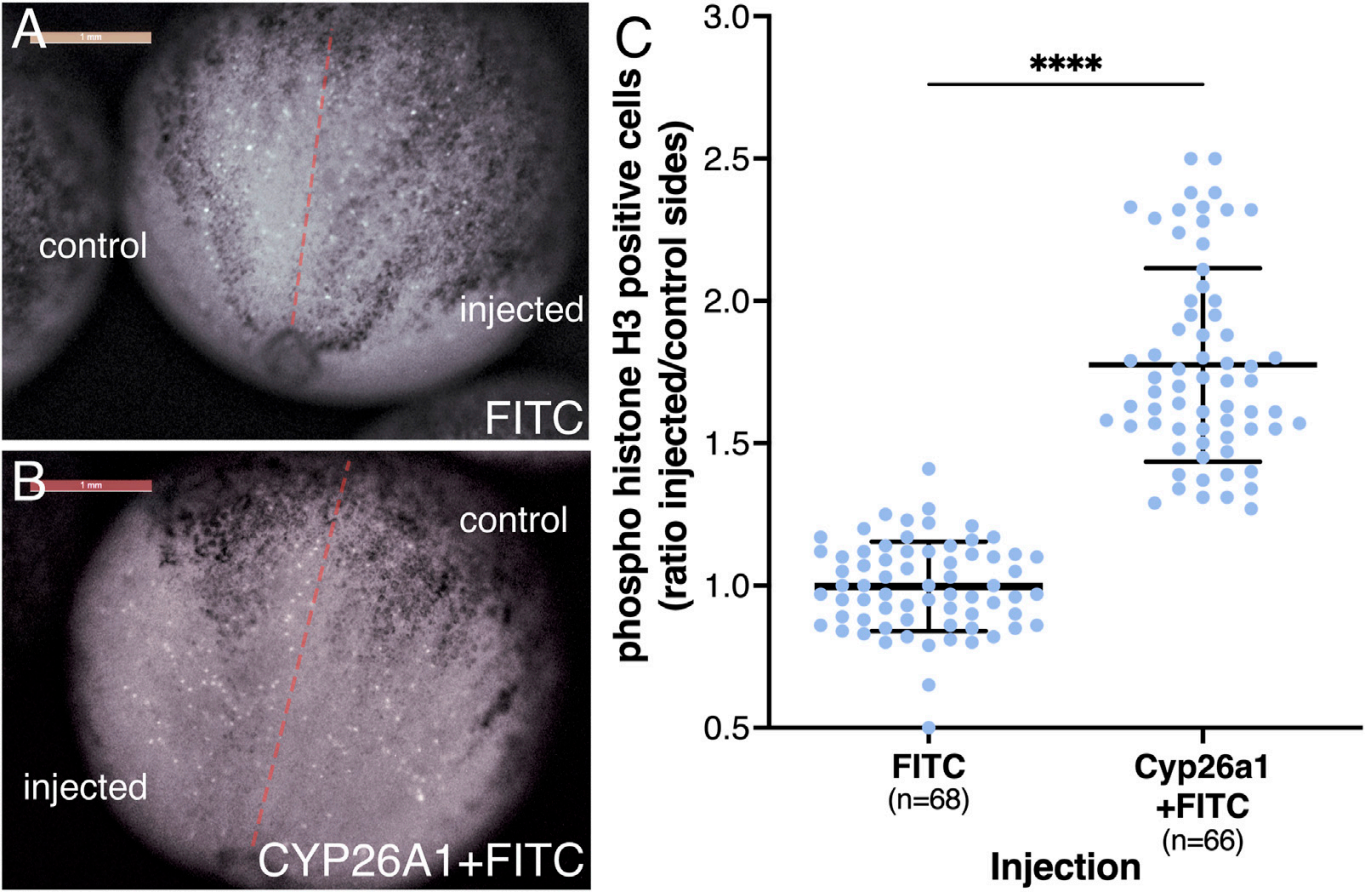
Reduced RA signaling promotes increased proliferation of neural precursors. Embryos were injected in one blastomere at the 2-cell stage to affect only half of the embryo. Injections included *cyp26a1* mRNA with FITC-dextran or FITC-dextran alone. At the onset of gastrulation, the embryos were sorted based on the injected side and further incubated until late gastrula. Embryos were stained by immunohistochemistry with antibodies against pHH3. The positive cells were counted on both sides of the midline, averaging 24 pHH3 positive cells on the control side in each embryo. **(A)** Control embryo injected with FITC-dextran on the right side. **(B)** Embryo injected with *cyp26a1* mRNA + FITC-dextran on the left side. **(C)** Relative proliferation analysis based on the number of pHH3-positive cells on both sides. The distribution of all embryos analyzed is shown. The number of embryos analyzed is shown. ****, *p* < 0.0001 compared to the control (FITC-dextran) sample ratio.

To study the effects of reduced RA on cell proliferation during neuroectodermal differentiation, we injected *cyp26a1* mRNA into one blastomere at the 2-cell stage promoting the reduction of RA on one side of the embryo. To identify the injected side, FITC-dextran was co-injected and, at the onset of gastrulation, embryos were sorted according to the injection side (left or right). The embryos were incubated to late gastrula (NF12.5) and subjected to cell proliferation analysis in the developing neural plate. To identify dividing cells, the embryos were stained for phosphorylated histone H3 (pHH3) (Prigent and Dimitrov, 2003), and the positive cells were counted on both sides of the midline (about 24 pHH3 positive cells/control side/embryo). As an additional control, embryos were injected on one side with FITC-dextran only. Analysis of the embryos revealed a clear increase in pHH3-positive cells on the *cyp26a1* RNA injected side (Figures 7A, B). For each embryo we calculated the ratio between the number of pHH3-positive cells on the injected side and the control side (Figure 7C). The results show that the reduction in RA signaling promotes an increase in cell proliferation of 1.77-fold compared to the control sides and control samples. The increased cell division level is in agreement with the gene expression changes observed in the early neuroectodermal regulatory network (Figure 5) and the expansion of the neural plate (Figure 6).

## Discussion

### Reduced RA signaling induces NTDs

Multiple reports have described a fetal retinoid syndrome (embryopathy) in humans that results in developmental malformations, including a wide range of central nervous system defects (Lammer et al., 1985; Rappaport and Knapp, 1989; Morrison et al., 2005; Autret-Leca et al., 2010). Retinoid-exposed human embryos display neural tube defects similar to those spontaneously observed in fetuses (Copp et al., 2013; Wolujewicz et al., 2021b; Engelhardt et al., 2022). Gene expression studies have also revealed a connection between excess RA and NTDs in humans, including increased RAR transcription in human anencephaly samples (Xiaolu et al., 2023) and frameshift mutations in CYP26A1 linked to NTD induction (Rat et al., 2006). However, the lack of NTD cases for study due to spontaneous abortions and medically terminated pregnancies in retinoid-exposed pregnancies due to malformations presents a challenge for accurate estimations (Altıntaş Aykan and Ergün, 2020; Cha et al., 2022; Abdelmaksoud et al., 2023). In multiple experimental models, excess RA induces NTDs, and treating embryos with excess RA is a common method for studying NTD induction (Diez del Corral and Morales, 2014; Li et al., 2015; Tonk et al., 2015; Murphy et al., 2021). These RA-treated embryos develop NTDs resembling human malformations (Li et al., 2015; Tonk et al., 2015; Maassel et al., 2021; Swann-Thomsen et al., 2021).

Multiple components of the RA metabolic and signaling network have been identified as potential contributors to the formation of neural tube closure defects in genetic studies of human NTD patients (Deak et al., 2005; Rat et al., 2006; Tran et al., 2011; Pangilinan et al., 2012; Pangilinan et al., 2014; Li et al., 2018; Zou et al., 2020; Wolujewicz et al., 2021a). These genetic variants (single nucleotide or copy number) could affect both regulatory elements, leading to changes in gene expression, or the activity of encoded proteins. Reduced activity resulting from some of these missense, frameshift, or regulatory variants raised the possibility that also reduced RA signaling can induce NTDs. In experiments using *Xenopus* embryos, we found that the inhibition of RA biosynthesis via its inhibitors (DEAB or citral) resulted in a high incidence of NTC defects. The types of closure defects induced in these experiments correspond to common neural tube closure malformations observed in humans. These inhibitors are widely used and known to inhibit retinaldehyde dehydrogenases in the embryo (Koppaka et al., 2012; Shabtai et al., 2018), indicating that reduced RA signaling can induce NTDs in developing embryos. We also used an enzymatic approach to reduce signaling levels, overexpressing the RA hydroxylase Cyp26a1 (Isoherranen and Zhong, 2019; Roberts, 2020), to complement the pharmacological inhibition of RA biosynthesis. Overexpression of Cyp26a1 also efficiently induced NTC defects. Injection of *cyp26a1* RNA into specific lineages showed that NTC defects were efficiently induced when RA levels were reduced in the prospective neuroectoderm but not when targeting the mesoderm. These findings support a normal role for RA signaling in the process of precise neural tube closure.

### EtOH exposure induces NTDs through a reduction in RA

NTDs have been described in a number of studies of alcohol-treated experimental animal models suggesting multiple etiological explanations (Randall and Taylor, 1979; Boschen et al., 2021; Chen et al., 2005; Rovasio and Battiato, 1995; Jaurena et al., 2011). Experimental evidence from a number of studies, including embryonic, molecular, and enzymatic experiments, has demonstrated that exposure to EtOH reduces RA signaling (Deltour et al., 1996; Yelin et al., 2005; Kot-Leibovich and Fainsod, 2009; Wang et al., 2009; Marrs et al., 2010; Ballard et al., 2012; Muralidharan et al., 2015; Rah et al., 2018; Shabtai et al., 2018; Shukrun et al., 2019; Gur et al., 2022a). This reduction in RA biosynthesis induced by EtOH causes a similar range of neural tube closure defects compared to RA-inhibited *Xenopus* embryos. Further analysis of the EtOH effect on *Xenopus* embryos showed that NTC defects were induced in a concentration-dependent manner and the types of NTC defects observed recapitulated the main defects described in humans. Human embryos exposed to alcohol develop FASD, which involves a wide array of developmental malformations, and behavioral, cognitive, and social deficiencies (Popova et al., 2023; Jones, 2022; May et al., 2022). Among them, some patients suffering from FAS, the severe form of FASD, also develop NTDs (Popova et al., 2016). Diverse additional malformations of the central nervous system are also common among FAS patients (Popova et al., 2016). Although NTDs are not common among FAS patients, they occur more frequently in this group of patients than in the general population. The induction of NTDs in humans and other vertebrate experimental models by EtOH exposure during embryogenesis, is likely due to the reduction in RA levels, with the lower incidence of severe NTD forms among FAS patients probably the result of embryonic lethality (Windham et al., 1997; Andersen et al., 2012; Luukkonen et al., 2023).

The EtOH-induced NTC defects resembled the defects induced by the RA biosynthesis inhibitors DEAB and citral. To demonstrate that also in the case of NTC defect induction, EtOH promotes a competitive reduction in RA signaling, RA or its biosynthetic precursors, ROL or RAL were employed to rescue the NTC defects (Blaner, 2019; Berenguer and Duester, 2022). Retinoid (RA, ROL, and RAL) supplementation of EtOH-treated embryos showed that this pharmacological manipulation significantly prevented the development of NTC defects thus supporting the alcohol-dependent reduction in RA levels. These retinoids have been previously shown to rescue other EtOH-induced developmental malformations in multiple experimental models (Graham and Ferm, 1985; Wang et al., 2009; Serrano et al., 2010; Ballard et al., 2012; Hwang et al., 2012; Sarmah and Marrs, 2013; Muralidharan et al., 2015; Gupta et al., 2016; Ojeda et al., 2018; Rah et al., 2018; Shabtai et al., 2018; Cadena et al., 2020). Together these observations further support the previous results showing that the main biochemical effect of EtOH clearance during embryogenesis is a reduction in RA signaling through competition with enzymes involved in the biosynthesis of RA (Grummer and Zachman, 1990; Deltour et al., 1996; Kot-Leibovich and Fainsod, 2009; Shabtai et al., 2018).

In order to understand the involvement of RA signaling in the NTC process, we took advantage of the pharmacological nature of our NTC defect induction experiments and added the RA inhibitors (EtOH or DEAB) at different developmental stages. NTC defect analysis as a function of the RA biosynthesis inhibition timing, revealed that the effect was evident up to early gastrula stages and that by mid-gastrula, this effect is marginal or absent. This observation was confirmed in timed rescue experiments with retinoids. In these experiments the RA inhibition was started during late blastula and retinoid addition was performed as a function of the developmental stage. Also in the rescue experiments, early gastrula showed the highest sensitivity for NTC defect induction by reduced RA signaling. The beginning of gastrulation marks the onset of RA signaling (Ang and Duester, 1999; Niederreither et al., 1999; Grandel et al., 2002; Gur et al., 2022b) and RA signaling onset follows the transcriptional activation of the retinaldehyde dehydrogenases, mainly *aldh1a2* but also *aldh1a3,* at the initiation of gastrulation in the embryonic organizer (Chen et al., 2001; Shabtai et al., 2018; Parihar et al., 2021; Gur et al., 2022a; Lupo et al., 2005). In agreement, early RA signaling localizes to the embryonic organizer as shown in multiple vertebrate models (Rossant et al., 1991; Hogan et al., 1992; Deltour et al., 1996; Yelin et al., 2005; Samarut et al., 2015). Thus, the early sensitivity window for NTC defect induction by reduced RA signaling suggests a very early event during late blastula or early gastrula, either in the embryonic organizer itself, in cells derived from this structure, or in the ectodermal cells induced to become neural, which are key players in the proper formation of the neural plate. Our results identified the prospective neuroectodermal cell as the main target of the RA signal.

### Reduced RA signaling affects the differentiation program of neural precursors

Formation of the neural tube begins with the induction of neuroectodermal precursors from the embryonic ectoderm by secreted proteins that block BMP and Wnt signaling (reviewed in Lee et al., 2014). These neuroectodermal precursor cells begin to express a number of transcription factors that stabilize their neural fate and promote their proliferation to become neural plate stem cells. Downregulation of the genes encoding *gmnn* and *foxd4l1* results in exit from the cell cycle and differentiation of the neural stem cells (Kroll et al., 1998; Sullivan et al., 2001). We showed that the developmental stage sensitivity for NTC defect induction by alcohol and other RA-reducing agents maps to the onset of gastrulation. At the onset of gastrulation in *Xenopus* embryos, cells from the Spemann-Mangold organizer involute and migrate rostrally forming the leading edge of the mesendoderm/prechordal mesendoderm (LEM/PCM) that secretes the anti-BMP and anti-Wnt signals necessary for neural induction (Kaneda and Motoki, 2012; Huang and Winklbauer, 2018; Winklbauer, 2020). These cells also express one of the retinaldehyde dehydrogenases, *aldh1a3*, and as a result, produce RA (Lupo et al., 2005; Gur et al., 2022b). We have recently shown that reduced RA signaling affects early gastrulation morphogenetic movements resulting in a transient delay in the rostral migration of the LEM/PCM (Gur et al., 2022a). Therefore, reduced RA signaling likely affects either the strength or timing of neural induction, or an early gastrula event in the neuroectodermal precursor cells, that will result in abnormal NTC.

To understand the involvement of RA signaling in neuroectodermal induction and in the early events in the formation of the neural plate, we analyzed changes in the expression of genes encoding the early neural transcription factors following RA levels manipulation (Lee et al., 2014). We observed an increased expression of *gmnn* and *foxd4l1.1* as a result of reduced RA signaling. These two transcription factors are activated by the initial neural induction and both downregulate the expression of *bmp4*, delay neural differentiation, and promote neural stem cell proliferation (Lee et al., 2014). In agreement, we observed increased proliferation in the neural plate and an actual expansion of this embryonic structure. Among the SoxB1 family members which maintain the neural stem cells in an undifferentiated state (Penzel et al., 1997; Mizuseki et al., 1998; Kishi et al., 2000), the effect of reduced RA appears more complex. Analysis of *sox3* revealed increased expression, thus suggesting an increase in the number of neural stem cells, while *sox2* exhibited no significant change. Neural plate formation and its subsequent closure to form the neural tube involves numerous signaling pathways and an extensive set of cellular behaviors and morphogenetic changes (Juriloff and Harris, 2018; Engelhardt et al., 2022). Disruption of any of these processes can lead to the formation of NTDs. Changes in cell proliferation in the neural plate can also affect the closure of the neural tube (Harris and Juriloff, 2010; Okae and Iwakura, 2010). Therefore, there is evidence supporting that increased neural stem cell proliferation can lead to the formation of NTDs. Although it is not clear what is the differentiation state of the neural stem cells, we can clearly conclude that decreased RA signaling affects the process of neural plate formation and differentiation, leading to NTD formation.

## Data Availability

The original contributions presented in the study are included in the article/Supplementary Material, further inquiries can be directed to the corresponding author.
