# Supplementary material for: Alcohol induces neural tube defects by reducing retinoic acid signaling and promoting neural plate expansion
Source: Front Cell Dev Biol. 2023 Dec 5;11:1282273. doi: 10.3389/fcell.2023.1282273 (PMC10728305; doi:10.3389/fcell.2023.1282273)
Supplement: Supplementary file 1 [file DataSheet1.PDF]

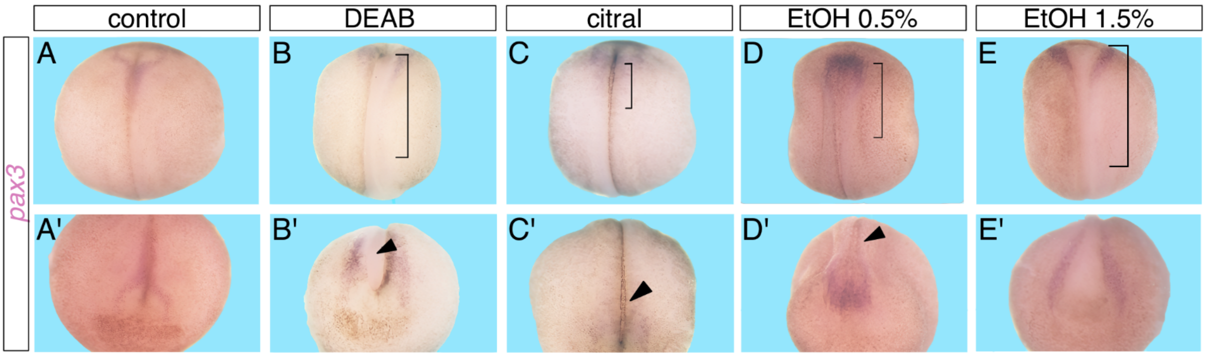

Supplemental Figure S1. Inhibition of RA biosynthesis induces neural tube closure defects. Inhibition of RA biosynthesis was induced by treatment with DEAB (60 μM), citral (50 μM), or EtOH (0.5% or 1.5% vol/vol). Treatments were initiated during mid-blastula stages (NF8), and embryos were allowed to develop to neural tube closure stages (NF19-20). Experimental and control embryos were processed for whole-mount *in situ* hybridization with the neural plate marker *pax3*. (A-E) Dorsal view of embryos, anterior to the top. (A′-E′) Anterior view of the same embryo shown in the panel above, dorsal to the top. Brackets mark NTC defects and arrowheads open neural tubes.

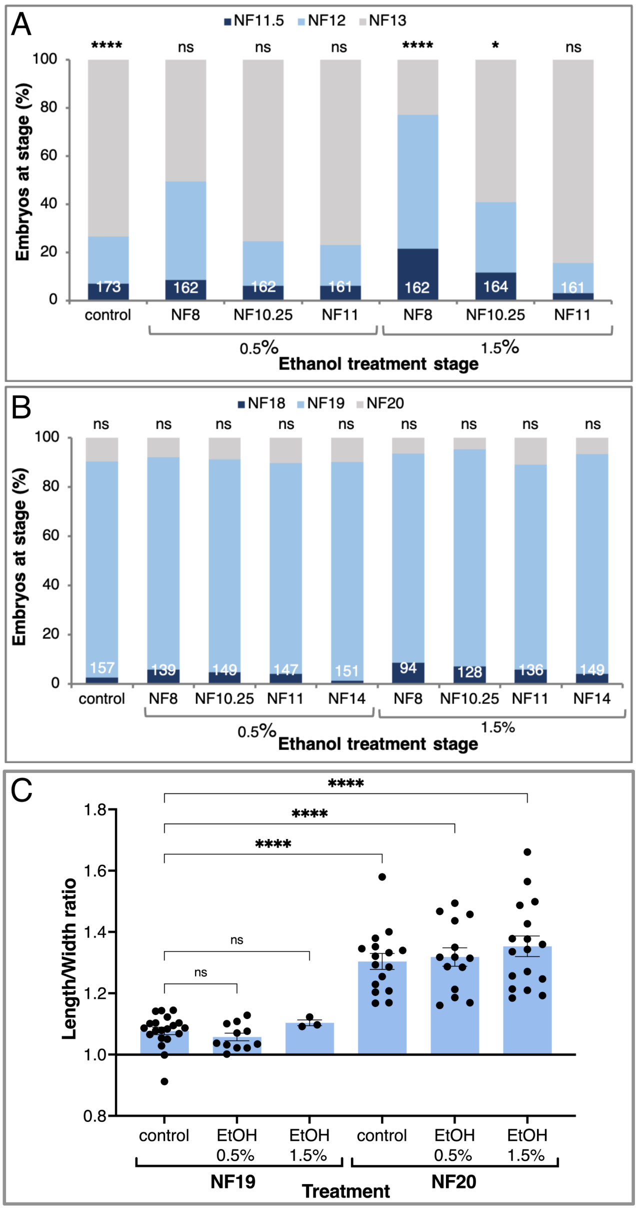

Supplemental Figure 2. Developmental progression as a result of EtOH exposure. (A, B) Embryos were treated during midblastula (NF8), early gastrula (NF10.25), mid gastrula (NF11), or early neurula (NF14) with low (0.5%) or high (1.5%) EtOH. The stage reached by the treated embryos was determined at the onset of neurula stages (NF13)(A) and neural tube closure stages (NF19-20)(B). The sample size and the percent embryos at each stage are shown. (C) Previously staged EtOH-treated or control embryos were measured to determine their length and width. The length-to-width ratio was calculated and plotted for EtOH-treated (0.5% and 1.5%) and control embryos for either NF19 and NF20. The ratio distributions are shown. *, $p<0.05$; ****, $p<0.0001$; ns, not significant.

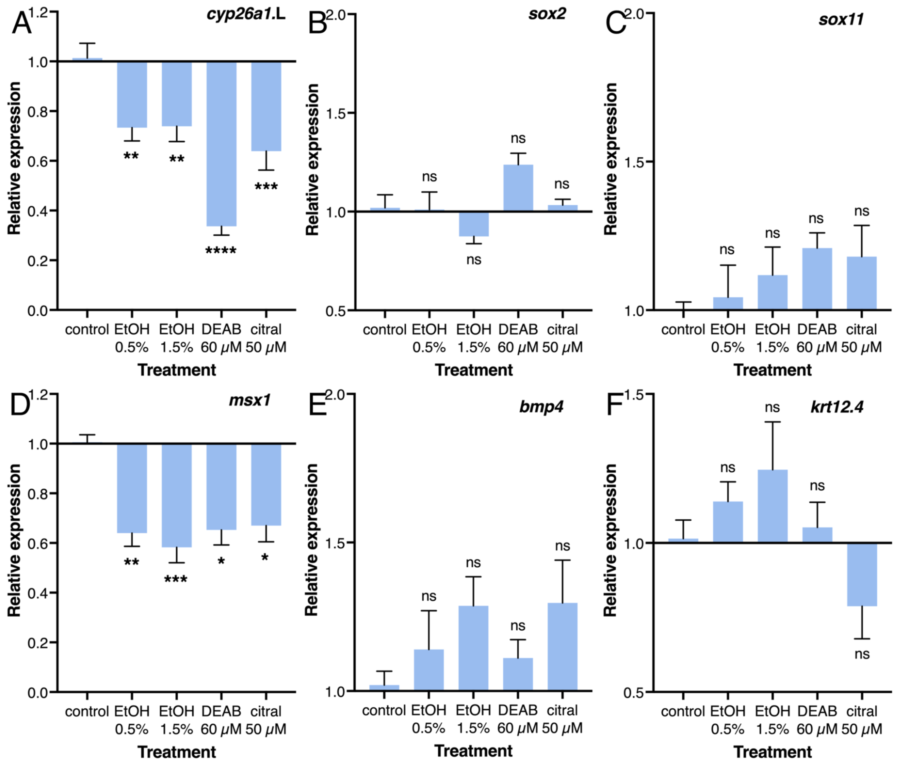

Supplemental Figure S3. RA is required for the regulated activation of the neuroectodermal transcriptional network. Embryos were treated to reduce the level of RA signaling by exposing them to EtOH, DEAB, or citral from blastula stages (NF8). RNA was extracted during early/mid gastrula (NF10.5), and the relative expression level of multiple components of the early neural differentiation network was determined by qPCR with gene-specific primers. *, p<0.05; **, p<0.01; ***, p<0.001; ****, p<0.0001; ns, not significant. Samples compared to the control sample.
